# Acute aortic dissection combined with cardiac tamponade in an elderly patient saved by pericardial drainage: A case report

**DOI:** 10.1002/ccr3.3513

**Published:** 2020-11-16

**Authors:** Kazunobu Une, Yui Hidaka, Junji Maeda, Hiroki Kinoshita, Hiroshi Kodama, Katsutoshi Sato

**Affiliations:** ^1^ Department of Emergency and General medicine JA Onomichi General Hospital Onomichi City Japan; ^2^ Department of Cardiology JA Onomichi General Hospital Onomichi City Japan; ^3^ Department of Cardiovascular Surgery JA Onomichi General Hospital Onomichi City Japan

**Keywords:** acute medicine, cardiovascular disorders, social care, social justice

## Abstract

Acute aortic dissection combined with cardiac tamponade is fatal. The radical treatment is an aortic replacement; however, the risk is high. We suggest conservative treatment with pericardial drainage as a treatment option in elderly patients with comorbidities.

## Graphical abstract

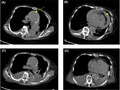

## 1 INTRODUCTION

A 94‐year‐old woman presented with acute aortic dissection combined with cardiac tamponade. Since the hemodynamics were unstable, surgical treatment was considered. However, due to her advanced age, dementia, and chronic kidney disease, pericardial drainage was urgently performed. Her clinical course continued steadily, and she was transferred on day 62.

In Japan, the aging population has been rapidly growing, and there have been more cases of elderly patients being transported by ambulance. Among the elderly population, acute aortic dissection (AAD) combined with cardiac tamponade has a high degree of urgency and severity, with aortic replacement being the radical treatment. However, the number of elderly patients who are difficult to treat surgically because of a decline in cognitive function and activities of daily living (ADL) has been increasing. Here, we reported a case of AAD with cardiac tamponade in an elderly patient who was treated by pericardial drainage (PD) and had a good clinical course.

## 2 CASE HISTORY/EXAMINATION

A 94‐year‐old woman presented to the hospital with persistent back pain and transient left hemiparesis. The back pain began on the morning of October X. A medical examination conducted by a family doctor revealed left hemiparesis and hypotension. Left hemiparesis improved in about 30 minutes, and no other neurological deficits were observed. After that, the patient was referred to our hospital.

On admission, she was alert, and no paralysis or dysarthria was observed. Furthermore, she had no fever, a respiratory rate of 22 times/min, oxygen saturation of 95% in room air, blood pressure of 90/68 mm Hg, and pulse rate of 112 beats/min with sinus rhythm.

Total blood cell counts showed an increase in white blood cells to 9900/μL, mild anemia, and a hemoglobin level of 9.1 g/dL. The platelet count was 10.5 × 10^4^/μL, showing a slight decrease. The coagulation test was almost normal, but the D‐dimer level was very high at 25.2 ng/mL. Biochemical examination showed no abnormalities in liver function, but the albumin level decreased to 3.0 g/dL, indicating malnutrition. Renal function also decreased as indicated by an urea nitrogen level of 43.9 mg/dL and creatinine level of 2.04 mg/dL. The HbA1c level was normal.

The patient had dementia and chronic kidney disease. Although ADL was relatively dependent, her athletic performance declined with age, and her clinical frailty score was at least 4.

## 3 DIFFERENTIAL DIAGNOSIS, INVESTIGATIONS, AND TREATMENT

**Figure 1:**
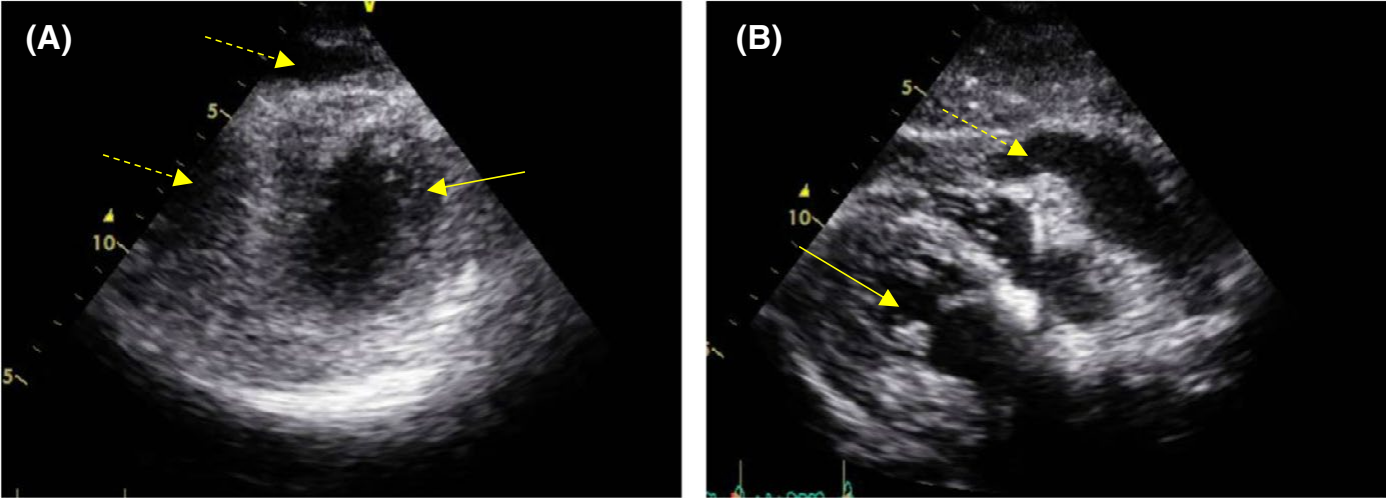
Echocardiography on admission. A, Image with the left ventricular short axis showing impaired RV filling due to cardiac tamponade. B, Image of the long axis of the left ventricle showing a large amount of pericardial effusion. ←, shows left ventricular; ⇠,shows pericardial effusion

**Figure 2:**
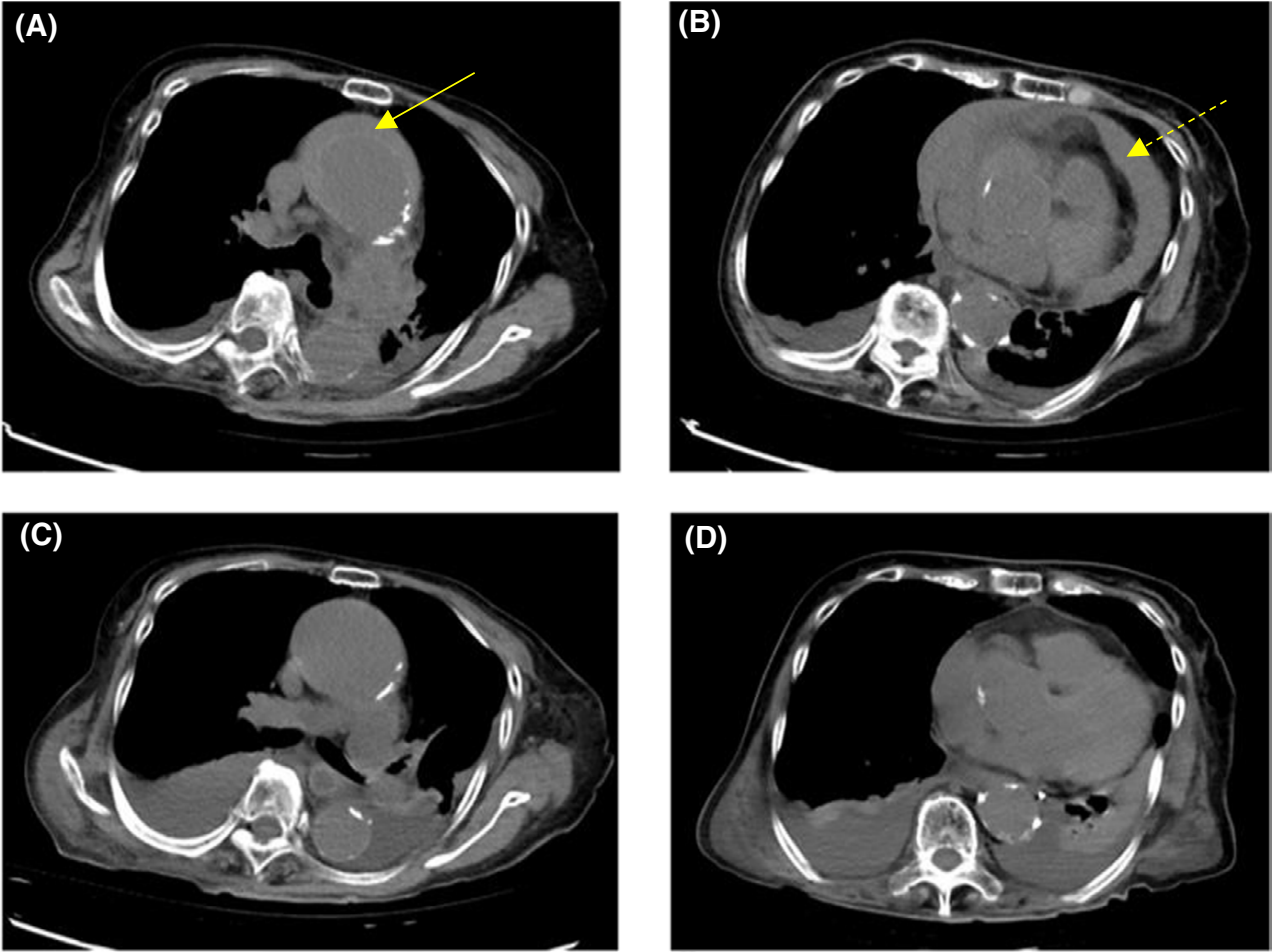
Non‐contract chest computed tomography (CT). A, B, Non‐contrast chest CT on admission shows a hematoma‐filled false lumen as a crescentic, high‐attenuation region following the aortic wall (crescentic high‐attenuation hematoma), and a pericardial effusion at the descending aorta level. C, D, Non‐contrast chest CT 8 days after admission shows absorption of the hematomas around the ascending aorta and reduction in the pericardial effusion. ←, shows high‐attenuation hematoma; ⇠,shows pericardial effusion

Based on the patient's blood pressure on arrival, a shock state and a cerebrovascular disorder were not strongly suspected. However, echocardiography revealed increased right ventricular pressure and pericardial effusion (Figure 1). She complained of back pain that was not strong but persistent, and transient neurological symptoms were documented; therefore, we suspected aortic dissection. We decided against the use of contrast medium because of the patient's renal function; therefore, non‐contrast computed tomography (CT) was performed without ECG gating to evaluate the structure of the aorta. CT showed a false cavity filled with hematoma along the aortic wall (Figure 2A–B); therefore, the patient was diagnosed with AAD combined with cardiac tamponade. Since the hemodynamics were unstable, surgical treatment was considered. However, due to factors such as the patient's advanced age, dementia, and chronic kidney disease, the perioperative risks were considered to be very high. We discussed the treatment policy with the patient and family members. We thought that aortic replacement may be fatal because it requires extracorporeal circulation, which will be an overly invasive procedure. Besides, even after surviving the surgery, the condition would be difficult to manage during the perioperative period, including during rehabilitation. Ultimately, with the consent of the patient and her family, we decided to urgently perform PD, monitor, and treat the circulatory state strictly without aortic replacement. Immediately, emergent PD was performed, blood pressure was monitored, and 30 mL of blood was drained twice. The heart rate decreased to 90 bpm, and systolic blood pressure rose to 110 mm Hg. After the patient was admitted to the intensive care unit, her blood pressure was controlled with a continuous antihypertensive drug under arterial pressure monitoring. PD was performed without an excessive increase in blood pressure, and the volume of drainage was 250 mL over 8 hours on the first day. After that, the volume of drainage decreased, and from the 2nd day, only ~ 30 mL/day of drained pale serum was observed. Pericardial effusion was evaluated by ultrasonic examination every day to confirm that there were no sudden increases.

## 4 OUTCOME AND FOLLOW‐UP

Rehabilitation for disuse prevention was started on the 3rd hospital day, and a CT scan was performed on the 8th day to confirm the condition. A decrease in pericardial effusion and a reduction in the dissection space of the ascending aorta were confirmed (Figure 2C–D). On the 13th day, a CT evaluation was performed again, and the patient's rehabilitation progress was favorable. She was then discharged from the intensive care unit. The course continued steadily thereafter, and she was transferred to a hospital for continual rehabilitation on the 62nd day. In a visit to our hospital 3 months after discharge, she was walking via a walker. No deterioration was observed by CT, and her clinical condition was good.

## 5 DISCUSSION

Gilon et al reported that 126 of 674 patients (18.7%) enrolled in the International Registry of Acute Aortic Dissection also had cardiac tamponade, which resulted in a 54% in‐hospital mortality rate. The mortality rate was more than double compared to that of AAD alone.^1^ In the present case, the patient was in a shock state on arrival with the possibility of a fatal course.

According to the United States guidelines,^2^ the number of PD procedures should be minimized, and PD should only be performed when circulation cannot be maintained before surgery because PD may increase the amount of fluid flowing into the pericardial space. Hayashi et al^3^ reported using a pigtail catheter to control the drainage volume and to prevent blood pressure from rising. In their study, PD was performed in 18 patients whose blood pressure dropped due to cardiac tamponade associated with AAD during the preoperative period. Circulatory dynamics were improved in 10 patients with a drainage volume of 30 mL, and the patients were then able to undergo operations. Fujii et al^4^ also reported the importance of controlling the drainage volume without causing the blood pressure to increase excessively. Honda et al^5^ proposed a PD volume of 10 mL while maintaining a blood pressure under 100 mm Hg in elderly patients older than ~80 years of age to avoid a drop in pericardial intracavitary pressure and a rise in blood pressure. They also reported that good results were obtained without surgery by appropriately repeating PD. As in our case, although a single drainage volume was 30 mL, a low drainage volume was intermittently obtained under strict blood pressure control and thereby good results were obtained. This provides an alternative treatment option for elderly patients with AAD combined with cardiac tamponade.

Hattori et al^6^ reported that the surgical treatment in nonagenarians can be successful, especially for those with a preoperative clinical frailty score ≤ 4. Aoyama et al^7^ also reported a comparative study on surgical and conservative treatment for patients with AAD who are older than 80 years of age in Japan. As a result, all in‐hospital mortality rates were significantly decreased in patients who underwent surgery. However, there was no significant difference in the event‐free survival rate when the presence or absence of complications was considered. As in our case, complications such as dementia, chronic kidney disease, and the age of 94 made it difficult to decide on a treatment method. According to our patient's ADL, the clinical frailty score was considered to correspond to at least 4, and her respiratory function was equivalent to 2 in the Hugh‐Jones classification, which indicated a mild dysfunction. When preoperative risk was calculated using Japan SCORE,^8^ the 30‐day surgical mortality rate was 54.5%, and the incidence of major complications was 87.0%. Since these rates were very high, we decided on a life‐saving strategy with lower invasiveness than surgical treatment. Therefore, PD was performed as one of the effective methods for releasing cardiac tamponade, even though it is not always recommended for patients with cardiac tamponade associated with AAD. As a result, the patient had a good clinical course, but the treatment method and family burden must be considered quickly during emergencies. In Japan, where the number of patients with complications is expected to increase due to further aging of the population, appropriate treatment decisions for emergent diseases based on the patient's background and more appropriate policies are needed. As a result, the accumulation of such cases will provide useful information for clinicians who encounter similar cases in the future.

## 6 CONCLUSION

With an aging population, the frequency of patients with highly severe and urgent needs is expected to increase. For elderly patients with AAD combined with cardiac tamponade, the treatment policy should be made carefully based on their ADL and comorbidities. Conservative treatment with PD should be considered as one of the options.

## 7 INFORMED CONSENT

Informed consent was obtained from the patient and family in the study.

## CONFLICT OF INTEREST

None declared.

## AUTHOR CONTRIBUTIONS

Une is the first author: managed the patient in the emergency department, and drafted the manuscript. Hidaka, Maeda, and Kinoshita: reviewed the manuscript and managed the patient in the intensive care department. Kodama and Sato: supervised the writing and submitting process. All authors discussed and approved the submitted manuscript.

## ETHICAL APPROVAL

All the procedures performed in this study were in accordance with the ethical standards of the institutional and national research committee and with the 1964 Helsinki Declaration and its later amendments or comparable ethical standards.

## Data Availability

The authors confirm that the datasets supporting the findings of this study are available from the corresponding author upon reasonable request.
